# Decoupling of Mechanical and Thermal Signals in OFDR Measurements with Integrated Fibres Based on Fibre Core Doping

**DOI:** 10.3390/s25041187

**Published:** 2025-02-15

**Authors:** Clemens Dittmar, Caroline Girmen, Markus Gastens, Niels König, Thorsten Siedenburg, Michael Wlochal, Robert H. Schmitt, Stefan Schael

**Affiliations:** 1 Physics Institute B, RWTH Aachen University, Templergraben 55, 52062 Aachen, Germany; 2 Fraunhofer Institute for Production Technology IPT, Steinbachstraße 17, 52074 Aachen, Germany; 3 Institute of Structural Mechanics and Lightweight Design, RWTH Aachen University, Templergraben 55, 52062 Aachen, Germany; 4 Laboratory for Machine Tools and Production Engineering (WZL), RWTH Aachen University, Templergraben 55, 52062 Aachen, Germany

**Keywords:** optical fibre sensor, OFDR, decoupling, temperature, strain

## Abstract

In this paper, a new measurement principle for decoupling mechanical and thermal signals in an OFDR measurement with integrated optical fibres is investigated. Previous methods for decoupling require additional measuring equipment or knowledge about the substrate properties. This new method is based solely on simultaneous measurements of two fibres with different temperature sensitivities resulting from different core doping processes. By exposing both fibres to the same thermal and mechanical load, the signal could be differentiated through the signal variations caused by the thermo-optical effect. The two fibres used in the tests have a sufficient response difference in the cryogenic temperature range. Therefore, the method is suitable for various applications, such as high-temperature superconductors as well as cryogenic and space applications.

## 1. Introduction

The practical properties of optical fibres, such as their small size, light weight, and immunity to electromagnetic interferences, have led to more and more optical fibres being used in telecommunications and measurement technology in recent decades [1,2,3,4]. The application areas are diverse and range from applications in telecommunications [5], where long transfer lengths benefit from low attenuation in the oil and gas and construction industries [6,7], where load data must be collected over very long distances, such as pipelines or tunnels. At the same time, fibre optic sensors are also being increasingly integrated for structural monitoring in smaller components, such as in lightweight construction [8] or in the energy storage sector [9,10]. One of the biggest advantages of optical fibres is their immunity to most external influences, making them ideal to withstand even harsh environments, delivering reliable data under various conditions. Especially in the field of aerospace [11,12] and when it comes to the cryogenic temperature range, fibre optic sensors have proven themselves to be suitable tools [13,14,15,16].

A distinction must be made between two main measuring principles: localised or quasi-distributed sensors, such as fibre Bragg gratings (FBGs) [17], which are based on wavelength-dependent backscattering from gratings imprinted in the fibre core, and distributed fibre-optic sensors, which are based on the principles of Rayleigh [18], Brillouin [19], or Raman scattering [20]. The distributed techniques have the advantage of using an unprocessed bare fibre, while fibres with FBGs need complex additional machining to be used as sensors. By exploiting the scattering of light within the fibre, distributed sensing systems can detect changes in parameters like temperature and strain with remarkable precision in the range of microstrain and 1 K [18,21]. This approach provides a continuous and spatially resolved view of the monitored environment, making it invaluable for applications such as structural health monitoring, environmental sensing, and industrial process control [22,23].

One main advantage of optical fibre sensors is their high sensitivity to various external influences, which makes them ideal as a fast and highly sensitive measurement solution [21]. But the most important advantage, i.e., being sensitive to various external influences, of fibre optic sensors is also a limitation. The high sensitivity of the fibres to various external influences such as strain and temperature leads to cross-coupling of the measurement parameters, so when several parameters influence the sensor, they can no longer be separated [24]. There are diverse solutions to overcome the limitations of cross-coupling. The first idea is to integrate additional sensors parallel to the optical sensor such as a second mechanically decoupled fibre [25], thermoresistors, or strain gauges. This method is not feasible for most use cases as it requires additional space and electrical wiring, eliminating the advantage of integrating thin and lightweight bare fibres. Another solution consists of measurement principles using specific fibres such as polarisation-maintaining (PM) fibres [26] or specifically designed FBGs such as the π-phase shifted FBGs [27] or custom-designed sensor arrays [28]. These custom-made fibres have the disadvantage of expensive and time-consuming pre-processing of the sensor fibres.

In addition, hybrid measurement systems that combine various sensor technologies based on different scattering principles, such as Brillouin and Rayleigh, have become increasingly important in structural monitoring. These systems integrate optical fibre sensors based on scattering techniques, fibre Bragg gratings, interferometric methods, and polarimetric approaches to enhance the accuracy and versatility of monitoring composite structures [24]. In Gorshkov et al. [29], a novel sensor is proposed to simultaneously measure strain and temperature changes within an optical fibre. It utilises Raman optical time-domain reflectometry and a wavelength-tunable quasi-monochromatic Rayleigh reflectometry system implemented through a microelectromechanical filter (MEMS). Also, systems based on a hybrid Raman–Brillouin scattering approach, leveraging multi-wavelength optical sources like Fabry–Perot (FP) lasers, are investigated in [30]. By employing a self-heterodyne detection scheme with a multi-wavelength optical local oscillator, these approaches fully use the advantages of FP lasers, enabling high-power Raman intensity measurements and precise detection of the Brillouin frequency shift parameter for each FP longitudinal mode.

In addition to the various advantages of these methods investigated so far, there are also some disadvantages, such as the additional use of electrical sensors or complex and expensive special fibres or hybrid measuring systems. In Ding et al. [31], a standard single-mode fibre (SMF) and a reduced cladding fibre are used to decouple thermal and mechanical signals due to their different responses to temperature and strain comparable to decoupling in FBGs. This enables the decoupling of thermal and mechanical signals in small temperature ranges with a linear correction, as many materials do not exhibit thermal expansion at cryogenic temperatures. There is practically no thermomechanical behaviour of carrier materials for the cryogenic range, and for larger measuring ranges, a polynomial dependency and the thermo-optical effect are necessary. This work uses this method to integrate two standard single-mode fibres with different core dopings in parallel as sensors. These are investigated using Rayleigh-based optical frequency domain reflectometry for the cryogenic temperature range. As described in [32], the developed method can determine the fibre-dependent parameters precisely for the required temperature range, which will be investigated in this study for a boron-doped fibre. The two fibres show the same response to mechanical loads but differ for thermal loads. This allows the mathematical decoupling of the two signals to measure strain and temperature individually with one system based on the thermo-optic effect, which has not been shown before in the cryogenic range. This enables the decoupling of thermal and mechanical load measured by distributed optical fibres in the cryogenic range.

## 2. Materials and Methods

The following section describes the mathematical background, the methods used, and the setup for thermal and mechanical decoupling.

### 2.1. Improved Spectral Shift Model

When the temperature during the optical frequency domain reflectometry (OFDR) measurement changes by a difference ΔT or a mechanical strain ε is induced, the refractive index and the length of the optical fibre change, causing a shift in the Rayleigh backscatter spectra. This spectral shift denoted by $\frac{\Delta \nu }{\hat{\nu }}$ is described by a linear model with Equation (1) [18].

(1) $$-\;\frac{\Delta \nu }{\hat{\nu }}\;=\;K_{T}\cdot \Delta T\;+\;K_{\varepsilon }\cdot \varepsilon$$

This describes a basic model for the changes of the spectral shift based on thermal and mechanical load with an error in the fibre parameters $K_{T}/K$ and $K_{\varepsilon }$ of approximately 10% [33].

The optical frequency shift of an optical fibre, which is embedded in a substrate, under thermo-mechanical stress can be determined more precisely with thermal and mechanical fibre parameters and the known thermo-mechanical expansion of the substrate. The spectral shift can be described by a thermal $F(T_{M},T_{R})$, a mechanical $K_{\varepsilon }\left(T_{M}\right)\cdot \varepsilon$, and a mixing term $G(T_{M},T_{R})$ [32].

(2) $$-\;\frac{\Delta \nu }{\hat{\nu }}\;=\;F\left(T_{M},T_{R}\right)\;+\;G\left(T_{M},T_{R}\right)\;+\;K_{\varepsilon }\left(T_{M}\right)\cdot \varepsilon_{\mathrm{External}}$$

$T_{M}$ is the measurement temperature and $T_{R}$ is the reference temperature. The mixing term describes the mechanical strain caused by the temperature change and the temperature dependence of the mechanical sensitivity coefficient $K_{\varepsilon }$ as described in Girmen et al. [32]

(3) $$G\left(T_{M},T_{R}\right)\;=\;K_{\varepsilon }\left(\frac{T_{M}\;+\;T_{R}}{2}\right)\cdot \varepsilon_{Sub.}\left(T_{M},T_{R}\right)$$

The purely thermal dependence can have higher polynomial orders in which the parameters depend on the doping of the fibre core.

(4) $$F\left(T_{M},T_{R}\right)\;=\;\int_{T_{R}}^{T_{M}}K_{T}\left(T\right)\;dT\;=\;A\cdot \left(T_{M}\;-\;T_{R}\right)\;+\;\frac{B}{2}\cdot \left(T_{M}^{2}\;-\;T_{R}^{2}\right)\;+\;O\left(T^{3}\right)$$

The mechanical sensitivity was assumed to increase linearly with the temperature [32,34].

(5) $$K_{\varepsilon }\left(T\right)\;=\;m\cdot T\;+\;b$$

The dependence of the thermal parameters on the fibre material, described by the thermo-optic coefficients [35], ensures that a variation of the doping in the fibre-optic core leads to a change in the spectral shift. This difference originates from the different band gap energies of the used materials and results in the fact that two fibres with similar core dimensions but different doping deliver different spectral shifts with the same pure thermal load. With mechanical loading, an equally significant shift is to be expected if the strain sensitivity is similar since the generation of this does not depend on the doping.

### 2.2. Two-Fibre Setup for Thermal–Mechanical Decoupling

The previous paragraph described the behaviour of a single fibre under thermal and mechanical load. At the same time, the following section uses the most important findings to enable thermo-mechanical decoupling through the use of two fibres. The following equations distinguish the two fibres by the index 1 or 2. Decoupling is achieved by analysing the difference in spectral shifts at the same location and time. Equation (6) therefore shows the difference between the fibre signals (1–2) on the left-hand side and the difference between the model parameters of the two fibres from Equation (2) on the right-hand side. To compensate for different strain sensitivities ($K_{\varepsilon }\left(T\right)$), the second fibre signal is rescaled by $\beta \left(T_{M}\right)\;=\;\frac{K_{\varepsilon ,1}\left(T\right)}{K_{\varepsilon ,2}\left(T\right)}$ so that the external strain influence is eliminated. The remaining difference between $G_{1}\left(T_{M},T_{R}\right)$ and $G_{2}\left(T_{M},T_{R}\right)$ needs to be significantly smaller than the difference between $F_{1}\left(T_{M},T_{R}\right)$ and $F_{2}\left(T_{M},T_{R}\right)$ in the measuring range discussed here to avoid any significant effect on the decoupling for smaller temperature differences and to neglect this part in Equation (6).

(6) $$\begin{array}{cc} -\;\frac{\Delta \nu_{1}}{\hat{\nu_{1}}}\;+\;\beta \left(T_{M}\right)\cdot \frac{\Delta \nu_{2}}{\hat{\nu_{2}}}\; & =\;F_{1}\left(T_{M},T_{R}\right)\;-\;\beta \left(T_{M}\right)\cdot F_{2}\left(T_{M},T_{R}\right)\\ & +\;G_{1}\left(T_{M},T_{R}\right)\;-\;\beta \left(T_{M}\right)\cdot G_{2}\left(T_{M},T_{R}\right)\\ & \approx \;F_{1}\left(T_{M},T_{R}\right)\;-\;\beta \left(T_{M}\right)\cdot F_{2}\left(T_{M},T_{R}\right) \end{array}$$

The left-hand part of the equation, which still has a direct temperature dependence due to the $\beta (T_{M})$ factor, is shifted to the right-hand side.

(7) $$\begin{array}{cc} -\;\frac{\Delta \nu_{1}}{\hat{\nu_{1}}}\; & =\;F_{1}\left(T_{M},T_{R}\right)\;-\;\beta \left(T_{M}\right)\cdot F_{2}\left(T_{M},T_{R}\right)\;-\;\beta \left(T_{M}\right)\cdot \frac{\Delta \nu_{2}}{\hat{\nu_{2}}} \end{array}$$

With the measured shift $\frac{\nu_{2}}{\hat{\nu_{2}}}$, the fibre parameters, and the Newton method for *x*-value determination, a temperature for the measured shifts is approximated. To summarise this, the right-hand side of Equation (7) is calculated for a set of possible measurement temperatures and the $T_{M}$ that leads to a value close to the value on the left-hand side of the equation is chosen. Through an iterative process, the deviation of the right and left sides of the equation is minimised until it is below the statistical uncertainty of the left value.

Two conditions must be considered to determine the strain component.

First: No measurable temperature change according to the method described above but fibre signal: Here, only external strain is present and this can simply be determined from Equation (2) with

(8) $$\varepsilon_{\mathrm{External}}\;=\;\frac{-\;\frac{\Delta \nu }{\hat{\nu }}}{K_{\varepsilon }\left(T_{M}\right)}$$

and $T_{M}\;=\;T_{R}$.

Second: A temperature change could be measured: In this case, the remaining signal is initially considered to be the strain that the measurement object exhibits. Assuming that the temperature change is smaller ($K_{\varepsilon }\left(\frac{T_{M}\;+\;T_{R}}{2}\right)\;\approx \;K_{\varepsilon }\left(T_{M}\right)$), the total strain can be calculated in the following way:

(9) $$\varepsilon \;=\;\varepsilon_{\mathrm{External}}\;+\;\varepsilon_{\mathrm{Sub}}\;=\;\frac{-\;\frac{\Delta \nu }{\hat{\nu }}\;-\;F\left(T_{M},T_{R}\right)}{K_{\varepsilon }\left(T_{M}\right)}$$

If the thermal expansion of the carrier material is also known, the measured strain can be resolved into the thermal and external components.

(10) $$\varepsilon_{\mathrm{External}}\;=\;\frac{-\;\frac{\Delta \nu }{\hat{\nu }}\;-\;F\left(T_{M},T_{R}\right)\;-\;G\left(T_{M},T_{R}\right)}{K_{\varepsilon }\left(T_{M}\right)}$$

The thermo-mechanical behaviour of the carrier material can, for example, be determined beforehand using active temperature changes.

(11) $$\varepsilon_{\mathrm{Sub}}\;=\;\frac{-\;\frac{\Delta \nu }{\hat{\nu }}\;-\;F\left(T_{M},T_{R}\right)}{K_{\varepsilon }\left(\frac{T_{M}\;+\;T_{R}}{2}\right)}$$

### 2.3. Introduction to Optical Frequency Domain Reflectometry (OFDR)

The optical fibre interrogator used for the proposed method was the Luna OBR-4613 system, developed by Luna Technologies (LUNA OBR) [18]. This system is an optical frequency domain reflectometer (OFDR) that relies on Rayleigh scattering for its operation. The measurement setup comprises a tunable laser source that emits light between 1260 nm and 1340 nm. The emitted light is divided into two paths using a coupler. One of these paths leads to the interferometric measurement setup, as illustrated in Kreger et al. [18], which is fully equipped for OFDR measurements. The light is introduced into the fibre under test (FUT) within the measurement arm, acting as the primary sensor. As the light travels through the FUT, it traverses the entire length of the optical fibre, and Rayleigh scattering and Fresnel reflections occur. Subsequently, the scattered and reflected light returns to the coupler, interfering with the reference arm’s light. The resulting interference pattern is detected by photodetectors.

As the tunable laser source’s wavelength is adjusted, it generates a periodic signal at the detector. The frequency of this signal depends on the position of the corresponding Rayleigh backscattering fibre segment. Employing Fourier transformation, the signal summation of all segments along the fibre is decomposed into its frequency components. Each frequency component corresponds to a specific location within the fibre, with the amplitude of each frequency component indicating the strength of the corresponding reflection. This backscattered signal can be treated as a characteristic fingerprint, staying the same for equal conditions.

### 2.4. Measurement Fibres Used for Setup

A boron-doped and a germanium-doped fibre were used in the measurement setup. These two fibres have the same core and coating diameter but different doping and cladding as listed in Table 1. The germanium-doped fibre was chosen as the standard telecommunication fibre with the required properties such as low attenuation and good availability. The boron-doped fibre offers the same transmission range and low attenuation with a difference in the thermal optical coefficient compared to the germanium-doped fibre. As described in Ghosh et al. [35], the thermal, optical coefficient is based on the band gap energy of the material and changes with different dopants in the fibre core, which enables decoupling of the thermal signals of the germanium and boron-doped fibres.

The coating of the fibres has a negligible influence on the strain sensitivity in the described setup, as the mechanically strongest material of a measuring body with integrated fibre dominates the strain, as shown in the simulations in Girmen et al. [32]. Both fibres are glued into the same narrow groove with Scotch-Weld 2216 Grey from 3M Germany to decouple the thermal and mechanical signal. This ensures that the position of the fibres in relation to each other does not change and both fibres experience the same mechanical and thermal load. One fibre is connected to the OBR device, and the other end of this fibre is spliced to the other fibre. This makes it possible to measure both fibres almost simultaneously, which avoids thermal or mechanical changes that would otherwise occur between the measurements.

**Figure 1:**
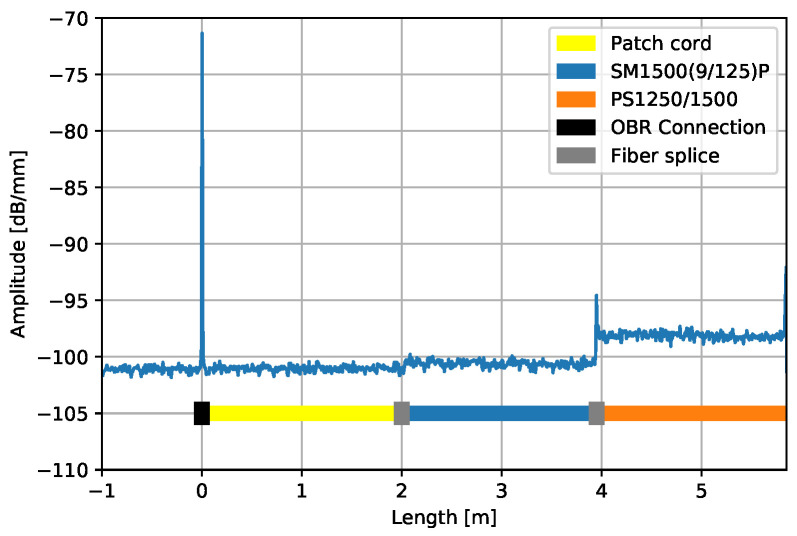
Shown is backscattering amplitude divided by distance versus distance from OFDR measurement. Signal of two different fibres, partly glued into aluminium structure, is shown.

Figure 1 shows the measured signal as the backscattered amplitude in dB over the fibre length in m. The signal from the boron-doped fibre shows a higher amplitude of the backscattered signal, resulting in a higher signal damping over long measurement ranges than the germanium-doped fibre. However, this effect does not yet affect measurements over the distance of 70 m that can be measured with the LUNA OBR [33].

The fibre parameters for the germanium-doped fibre were determined in a previous study and are listed together with the results of the boron fibre parameters in Section 3 in Table 2 and Table 3. Due to an inconsistent parameter definition in Girmen et al., parameters for $K_{T}\left(T\right)$ were used, which reproduce the curve in the publication [32] and are defined according to Equation (4).

The fibre parameters for the boron-doped fibre were determined similarly to the procedure described in [32]. The experimental setups for determining the strain and temperature sensitivity are analogous, and the measurements were carried out using the same measuring electronics. This is why the determination is not described in more detail here.

### 2.5. Setup for Signal Decoupling

**Figure 2:**
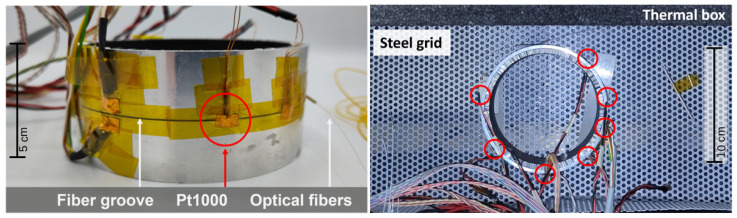
Ring-shaped test sample with two optical fibres in warm-up thermal box. Positions of Pt1000 are marked with red circle.

To test the decoupling with two fibres, a test ring sample was developed where both fibres were glued into a groove (0.5 mm depth and a width of 0.8 mm) of a cylindrical Al-6060 test body with dimensions of 50 mm height, 5 mm thickness, and a diameter of 110 mm. Both fibres were fed in and out of the ring at the same position. The test body also had a thin aluminium honeycomb structure inside, which did not affect the thermo-mechanical behaviour, as confirmed by additional studies which are not part of this work. The germanium-doped fibre was connected to the OBR system via a patch cable and connected via a splice to the boron-doped fibre. Pt1000 sensors were attached to the groove at six equidistant positions for reference temperature measurement. The experimental setup can be seen in Figure 2.

The aluminium ring was cooled down to 77K in a thermal box with liquid nitrogen, which was then thermally sealed. While warming up, the OBR system was measured continuously once per minute until room temperature was reached. A total of 1200 measurements were taken during the warm-up time of 18 hours. For measurements in the higher temperature range, the ring sample was step-wise heated in a climate chamber (Binder MK240/CTS 70-1500) from 233.15K to 353.15K. OBR measurements were recorded when stable temperatures were reached.

## 3. Results

In the following, the determined fibre parameters for the fibre doped with boron and the results of the double fibre measurement in cryogenic temperatures are shown.

### 3.1. Fibre Parameters

Table 2 shows the strain sensitivity for the boron fibre and for the germanium fibre.

Since the strain sensitivities lie within their uncertainties in the entire measurement range and are very similar in value, the approximation in Equation (6) is justifiable, as theoretically, the maximum contribution to the total differences in signal height at 100K, 200K, and 353K with reference at 77K are 0.08%, 0.5%, and 1.6%. In addition, from the similar spectral shift (maximum deviation $20\times 10^{-6}$ at $1000\times 10^{-6}$ external strain) of germanium fibre and boron fibre under variable external strain and constant temperature, it can be concluded that both fibres experience the same mechanical stress in the fibre core within the measurement uncertainty.

**Figure 3:**
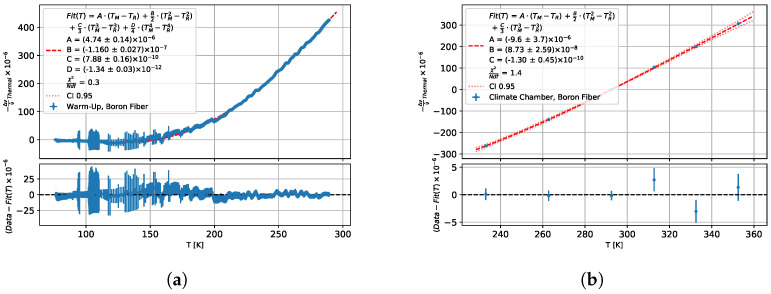
Thermal fibre signal from warm-up measurements of boron-doped fibre (**a**) for 77–290 K and (**b**) for 223–353 K against measurement temperature with fit of Equation (4).

For the temperature sensitivity of the boron-doped fibre, a fourth-order polynomial was chosen for the temperature range 77–290 K and a third-order polynomial was selected for the temperature range 223–353 K. The results are visible in Figure 3a,b.

**Figure 4:**
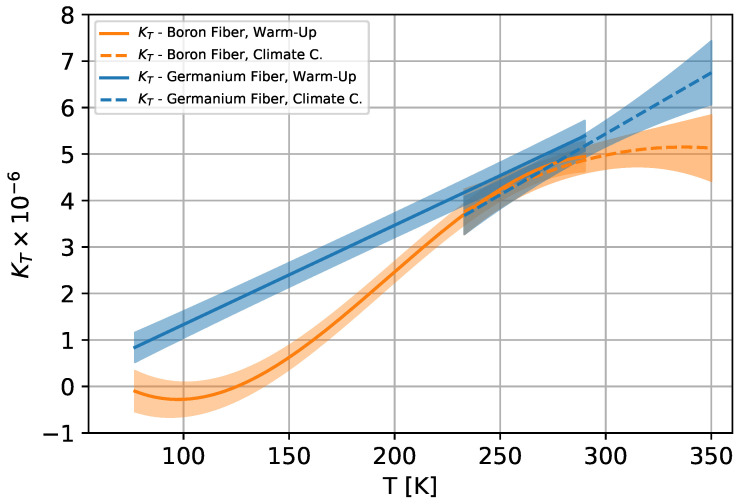
Temperature sensitivity for boron- and germanium-doped [32] single-mode fibre in temperature range 77 K–353 K.

The temperature sensitivity K(T) of the boron-doped and germanium-doped fibre is shown in Figure 4 and the parameters are given in Table 3. It can be seen that the sensitivity differs significantly in the range of 77–225 K and slightly between 300–350 K. For the sections of different temperature sensitivity, decoupling thermal and mechanical signals within the fibres is possible.

**Figure 5:**
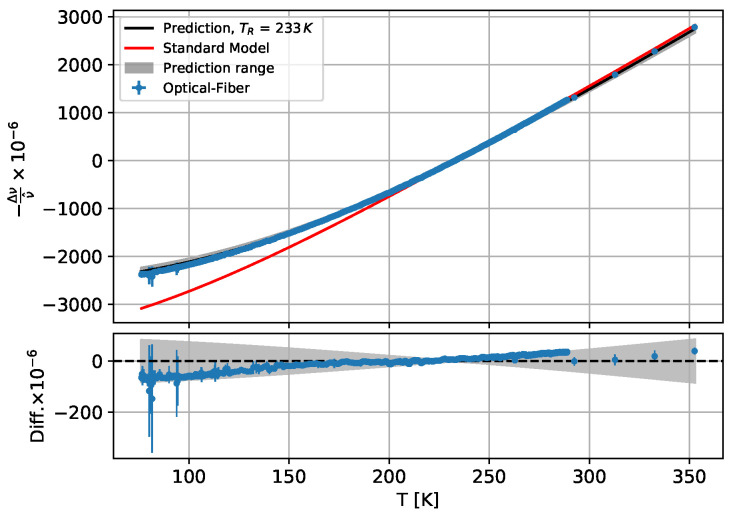
Standard model, measured and calculated spectral shift for the boron-doped fibre from the aluminium ring sample. The lower plot shows the difference between the data and the prediction.

The measured data, i.e., an average value directly at one of the PT1000 sensors, of the boron-doped fibre of the ring sample for the range 77–353 K are shown in Figure 5 together with the respective predictions calculated with Equation (2) and the thermal expansion parameters from Girmen et al. for the Al6060 carrier material and a reference temperature of 233 K.

It can be seen that the measured data lie within the uncertainty of the prediction model used, but a clear trend is visible, which will be discussed in Section 4. The effect of temperature determination from the systematic deviation of the boron fibre data on the model is discussed further in the following analysis of signal decoupling. However, the standard model does not describe the data curve in the lower temperature range.

### 3.2. Characterization of Decoupling Method

**Figure 6:**
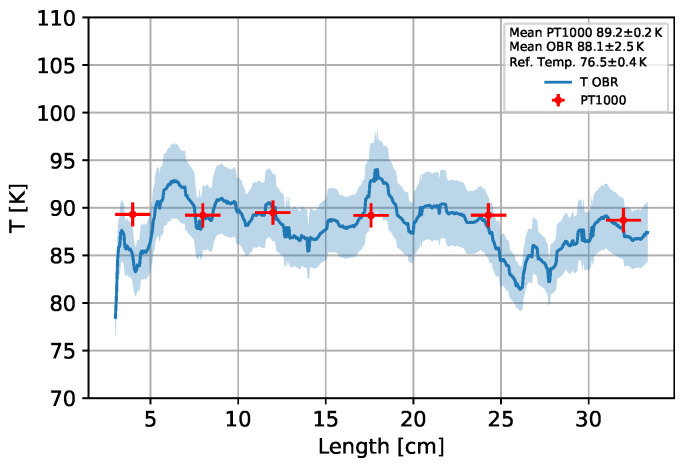
Temperature profile of the ring sample measured with the fibres by a 12 K shift. The temperatures measured by the six Pt1000 sensors are also shown.

Figure 6 shows the temperature profile of the ring and the Pt1000 data for a temperature difference of about 12K based on the measured fibre data and the decoupling method described. As the average critical temperature of HTS tapes is approximately 89 K, the temperature range from 78–110 K is investigated [36]. Figure 6 shows that the profile fluctuates within 5K, but the temperature is similar to the Pt1000 within a three-sigma environment. It could be detected that the germanium-doped fibre has a higher noise level than the boron-doped fibre. The blue area shows the statistical uncertainty on the temperature propagated from the statistical uncertainty for each fibre measurement point.

**Figure 7:**
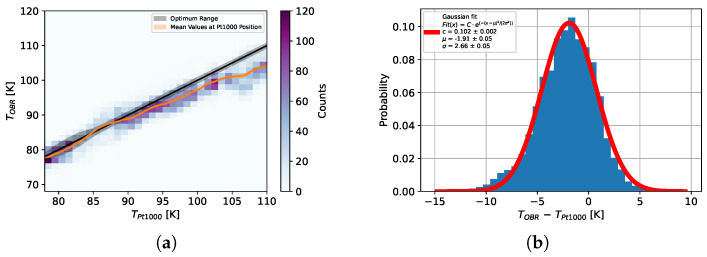
(**a**) A 2D histogram of the temperature determined with the fibres against the average Pt1000 temperature. (**b**) Differences between the fibre temperature and the mean Pt1000 temperature in each horizontal bin.

In Figure 7a, each fibre temperature over the ring section of the fibre signal is plotted in a 2D histogram against the average Pt1000 temperature.

The colour scale of the 2D histogram shows how flat the temperature profile is, i.e., how large the proportion of the temperature profile is within a temperature range of 1 K. As a result, only the resolution for dynamic temperature measurements is shown in Figure 7b. The resolution at a constant temperature (with measurement and reference temperature equal) over time in the cryogenic range is 1.2 K.

It can be seen that the mean fibre temperature follows the mean Pt1000 temperature for the range 78–90 K with a mean difference of −0.4K. However, from around 90 K, the fiber temperature exceeds this difference up to −5K. It was also observed that for larger temperature differences, the fibre temperature is lower than the Pt1000 temperature. This can be explained by the fact that the determined fibre parameters lead to a systematic deviation between the boron fibre signals and the theoretical prediction, which is still within the predicted range (see Figure 5). This systematic shift is consequently also observed in the temperature calculation. Therefore, the mean deviation can be considered a systematic error due to imperfectly determined fibre parameters when it came to temperature, which had a theoretical predicted max. of 7 K at 110 K. This system issue can be addressed in future calibration measurements. However, this is not necessary for an initial verification of the measurement solution. Overall, the histogram (Figure 7b) shows a Gaussian distribution with a mean offset of −1.9K and a spread of 2.7K. This demonstrates the effectiveness of the decoupling method for integrated fibres subjected simultaneously to thermal loads and mechanical stresses arising from the thermal expansion of the carrier material. Taking into account the now-defined fibre parameters and an estimate of the statistical noise in individual measurements, the uncertainties regarding the temperature to be determined and the strain calculated from this can also be estimated for other measurement materials.

**Figure 8:**
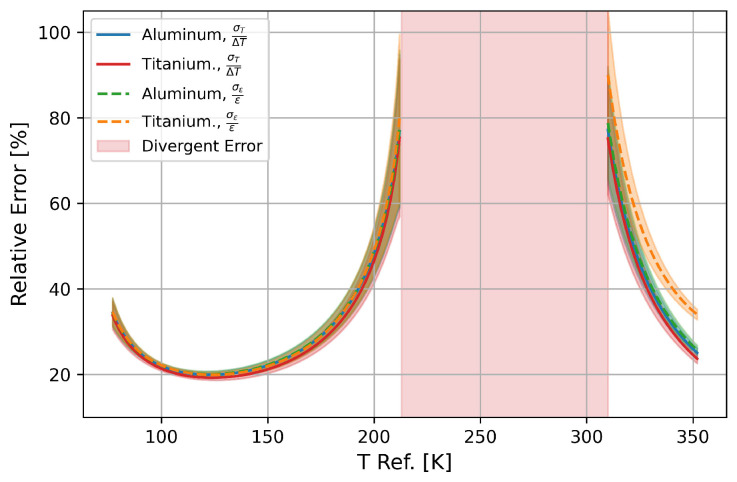
Calculated mean relative error for differential variables $\Delta T\;=\;T_{R}\;-\;T_{M}$ and strain for 0–10 K difference range compared to reference temperature, which is indicated on x-axis (specified for aluminium and titanium). Red area indicates that theoretical average error for measurements between 0 and 10 K is greater than measured value. Decoupling is therefore not possible in this area.

Figure 8 shows the mean relative error on the temperature difference (reference to measurement temperature) and the relative error on the strain for aluminium and titanium against the reference temperature. The uncertainty bands indicate the scatter. In addition, the relative uncertainties were not calculated in the range from 210K to 310K as the relative error diverges therein.

It can be observed that the relative errors between 77 and 210 K are the same within the variation. Only in the higher temperature range is the relative error on the strain for titanium about 10% larger. It can be assumed that since titanium has a significantly smaller thermal expansion than aluminium, the relative errors for most other metals that have a thermal expansion between aluminium and titanium are the same or in between the two. Furthermore, it can also be observed that where $K_{T}$ differs greatly, as shown in plot (Figure 4), the relative error is also minimised. This effect increases with higher reference temperatures, as the relative proportion of the temperature in the fibre signal increases.

## 4. Conclusions and Outlook

In this paper, the fibre parameter for boron-doped fibres was determined and compared to a germanium fibre. They showed a difference in the temperature-dependent coefficient for the range of 77–225 K and over 300K. This difference can be explained with the different thermal band energy behaviour of the core materials and described using the thermo-optical effect. The use of liquid nitrogen for the experiments with a temperature of 77 K caused the investigated limit of possible decoupling to be 77 K. The upper limit in this study was limited by the properties of the coating material and its resistance to high temperatures. This phenomenon was used to decouple the signal from thermal and mechanical stress by using two standard optical fibres in one component and thus enables a mathematical decoupling of the two signals from temperature and strain. By choosing a different doping for the second fibre, the temperature coefficient changes and thus can enable temperature decoupling. This method offers the advantage of decoupling strain and temperature in integrated optical fibres for setups where integrating a second loose fibre is not practical due to limited space, vacuum conditions, or high safety requirements.

This decoupling of mechanical and thermal loads was demonstrated for an aluminum body in the cryogenic temperature range. It could be shown that the method presented in this first investigation has a statistical resolution for dynamic temperature measurements of 2.7K in the range of 77–110 K with a maximum system deviation of −1.9K. In addition, it is possible to increase the accuracy significantly through multiple measurements and to eliminate the systematic deviation through a calibration measurement. With the calculated temperature, it is therefore possible to identify and calculate the remaining signal as mechanical. However, for an independent determination with higher resolution, the strain sensitivity also has to differ significantly.

It should be noted that the temporal resolution of different fibres with different cladding materials can be altered by different thermal conductivities. However, as only slow temperature changes are considered in this investigation, this possible effect is not relevant but should be investigated more in detail for faster temperature changes. The increasing noise for higher temperature changes can be addressed in further studies by using an optimised signal processing algorithm [37] or additional analysis methods such as a running reference approach [38]. However, these were not dealt with in this study as the focus was on the measurement method with two standard fibres for structure monitoring in the cryogenic range. Since the decoupling of thermal and mechanical signals is now possible without special fibres or additional measurement equipment using the method presented here, our method can be used, for example, in quench detection when it comes to high-temperature superconducting magnets such as those intended for the AMS-100 project [39,40]. The method is especially well suited to monitoring cryogenic applications with tubular or ring geometries such as superconducting magnets or accelerator structures. By embedding the fibre, the need for costly additional protection or structural integration required for free fibres is eliminated, enhancing the practicality and reliability of the method in such applications.

## Figures and Tables

**Table 1:** Fibre used for decoupling described by construction diameter and characterising coating materials.

Fibre Name	Core Doping	Core/Cladding/Coating Ø [μm]	Coating Material
SM1500(9/125)P	Germanium	9/125/157	Polyimide
PS1250/1500	Boron	9/125/145	Acrylate

**Table 2:** Strain sensitivity for germanium-doped [32] and boron-doped fibre with overall uncertainty for temperature range 77–300 K.

Fibre	$K_{\varepsilon }$ (T)	$\sigma_{K_{\varepsilon }}$
Germanium	$1.72\times 10^{-4}T\;+\;0.7775$	0.0052
Boron	$1.64\times 10^{-4}T\;+\;0.7789$	0.0053

**Table 3:** Temperature sensitivity parameters for germanium-doped [32] and boron-doped fibres within temperature range of 77–353 K. Uncertainties are given on $K_{T}$, and sum of squares of errors is used as absolute error for parameters.

	Germanium 77–290 K	Germanium 233–353 K	Boron 77–290 K	Boron 233–353 K
A$\cdot 10^{6}\;$[K^−1^]	−0.805	−2.44	4.74	−9.6
B$\cdot 10^{8}\;$[K^−2^]	2.14	2.63	−11.6	8.73
C$\cdot 10^{10}\;$[K^−3^]	-	-	7.88	−1.3
D$\cdot 10^{12}\;$[K^−4^]	-	-	−1.34	-
$\sigma_{stat}$	0.01	0.06	0.01	0.10
$\sigma_{sys}$	0.26	0.35	0.27	0.34

## Data Availability

Data underlying the results presented in this paper are not publicly available at this time but may be obtained from the authors upon reasonable request.
